# The Editor's Letter-Box

**Published:** 1920-11-06

**Authors:** 


*To the Editor of* The Hospital.

Sir,—"The more women as well as men who share Oxford's 'cloistral virtues' the better." So thinks the *Daily Mail*. Whether rightly or wrongly, Oxford herself will be able to judge in a few years' time.

At first sight those who would rather that Cambridge did not follow Oxford's example in granting degrees to women may appear unreasonable, for, since the students of Girton and Newnham attend the University lectures and sit for the various examinations, it would seem only logical to give them a recognition of their work. But that is not the real issue.

If women are to receive degrees, the number of female students will certainly increase enormously, new Colleges will be built or the present ones greatly enlarged, and in time there will doubtless be as many women as men in residence, if, indeed, not more. That prospect is not very attractive to those of us who know Cambridge, and have a little knowledge of feminine psychology.

That there will follow disputes between the male and female elements seems inevitable; one can picture, without, I think, being unduly pessimistic, a revival of the Suffragette type of disturbance, and the eternal "town" and 'Varsity feud will probably be replaced by one between petticoats and trousers. Young women *en masse* are more unreasonable even than they are singly, and young men, when fired by the prospect of a rag, are apt to forget the proprieties and to lose their sense of chivalry. And it must be remembered that the "popularising" of the two great Universities will mean that girls of very different types and classes must be catered for, and the authorities will have to be prepared to face problems which never present themselves under existing conditions. I therefore think that Cambridge will be well advised to watch the result of the experiment at Oxford before risking a move, the consequences of which may prove interesting, but are likely to lead to chaos and, perhaps, disaster.—Yours, etc., M.D. (Cantab.).

